# Development of an artificial intelligence system to indicate intraoperative findings of scarring in laparoscopic cholecystectomy for cholecystitis

**DOI:** 10.1007/s00464-024-11514-2

**Published:** 2025-01-21

**Authors:** Hiroki Orimoto, Teijiro Hirashita, Subaru Ikeda, Shota Amano, Masahiro Kawamura, Yoko Kawano, Hiroomi Takayama, Takashi Masuda, Yuichi Endo, Yusuke Matsunobu, Ken’ichi Shinozuka, Tatsushi Tokuyasu, Masafumi Inomata

**Affiliations:** 1 Department of Gastroenterological and Pediatric Surgery, Faculty of Medicine, Oita University, 1-1 Hasama-Machi, Yufu, Oita 879-5593 Japan; 2 Department of Information System and Engineering, Faculty of Information Engineering, Fukuoka Institute of Technology, Fukuoka, Japan

**Keywords:** Artificial intelligence system, Laparoscopic cholecystectomy, Acute cholecystitis, Surgical difficulty, Bile duct injury

## Abstract

**Background:**

The surgical difficulty of laparoscopic cholecystectomy (LC) for acute cholecystitis (AC) and the risk of bile duct injury (BDI) depend on the degree of fibrosis and scarring caused by inflammation; therefore, understanding these intraoperative findings is crucial to preventing BDI. Scarring makes it particularly difficult to perform safely and increases the BDI risk. This study aimed to develop an artificial intelligence (AI) system to indicate intraoperative findings of scarring in LC for AC.

**Materials and methods:**

An AI system was developed to detect scarred areas using an algorithm for semantic segmentation based on deep learning. The training dataset consisted of 2025 images extracted from LC videos of 21 cases with AC. External evaluation committees (EEC) evaluated the AI system on 20 cases of untrained data from other centers. EECs evaluated the accuracy in identifying the scarred area and the usefulness of the AI system, which were assessed based on annotation and a 5-point Likert-scale questionnaire.

**Results:**

The average DICE coefficient for scarred areas between AI detection and EEC annotation was 0.612. The EEC’s average detection accuracy on the Likert scale was 3.98 ± 0.76. AI systems were rated as relatively useful for both clinical and educational applications.

**Conclusion:**

We developed an AI system to detect scarred areas in LC for AC. Since scarring increases the surgical difficulty, this AI system has the potential to reduce BDI.

**Supplementary Information:**

The online version contains supplementary material available at 10.1007/s00464-024-11514-2.

Laparoscopic cholecystectomy (LC) is the standard procedure for cholelithiasis and acute cholecystitis (AC) [1, 2]. In LC, the most critical clinical consideration is prevention of biliary duct injury (BDI). BDI is the most common and serious intraoperative complications of cholecystectomies. It is often very morbid and leads to additional surgery, prolonged recovery time, higher medical costs, and reduced quality of life [3]. Despite the advancements in techniques, the prevalence of BDI has remained relatively unchanged for an extended period, with estimates ranging from 0.4 to 0.6% [4, 5]. To prevent BDI in LC, we developed artificial intelligence (AI) systems capable of indicating anatomical landmarks in the appropriate surgical phase and assessing the achievement of safe surgery during LC [6–8]. We conducted clinical feasibility trials to assess the landmark detection performance of AI systems and objectively demonstrate their usefulness [9, 10]. However, these AI systems have not demonstrated sufficient performance in preventing BDI in cases of AC with severe inflammation.

The difficulty of LC for AC largely depends on the degree of fibrosis and scarring of the tissue caused by inflammation, with greater fibrosis and scarring leading to increased surgical difficulty and a higher risk of BDI [11]. Therefore, surgeons must define surgical difficulty based on intraoperative findings during LC for AC and 25 intraoperative findings have been proposed to determine this difficulty [12]. Among these, the intraoperative finding of scarring has a particularly significant impact on surgical difficulty. Scarring is a condition in which the layered structure of the gallbladder wall is disrupted owing to a high degree of fibrosis caused by inflammation. These conditions make dissecting and exposing the layers required for safe LC impossible [13]. In the surgical difficulty grading system based on intraoperative findings [14], scarring of Calot’s triangle area is classified as highly difficult. Specifically, diffuse scarring in this area is classified as the highest level of surgical difficulty, warranting bailout surgery as suggested in the Tokyo Guidelines 18, such as fundus first technique, subtotal cholecystectomy, and open conversion to prevent BDI [11, 14]. Intraoperative findings of scarring can assist in the prevention of BDI. However, this requires the tacit knowledge of an expert surgeon with extensive experience. Therefore, young surgeons with limited experience or those from medically underdeveloped countries may find it challenging to accurately diagnose the intraoperative findings of scarring. Failure to diagnose appropriate range of scarring could lead to the occurrence of BDI during LC for AC. If something could indicate intraoperative findings of scarring during LC of AC, it would be useful for the prevention of BDI. This study aimed to develop and evaluate an AI system to detect an intraoperative finding of scarring during LC.

## Materials and methods

### Development of AI system

We have developed AI systems based on the concepts of visualizing the tacit knowledge of expert surgeons [6, 9, 10]. The development process of AI system involves collecting video data of adequate surgical cases, extracting still images from the video data and having expert surgeons annotated the tacit knowledge directly onto these images. These annotated images serve as training datasets for the AI to learn and improve this performance.

#### Preparations of datasets

**Fig. 1:**
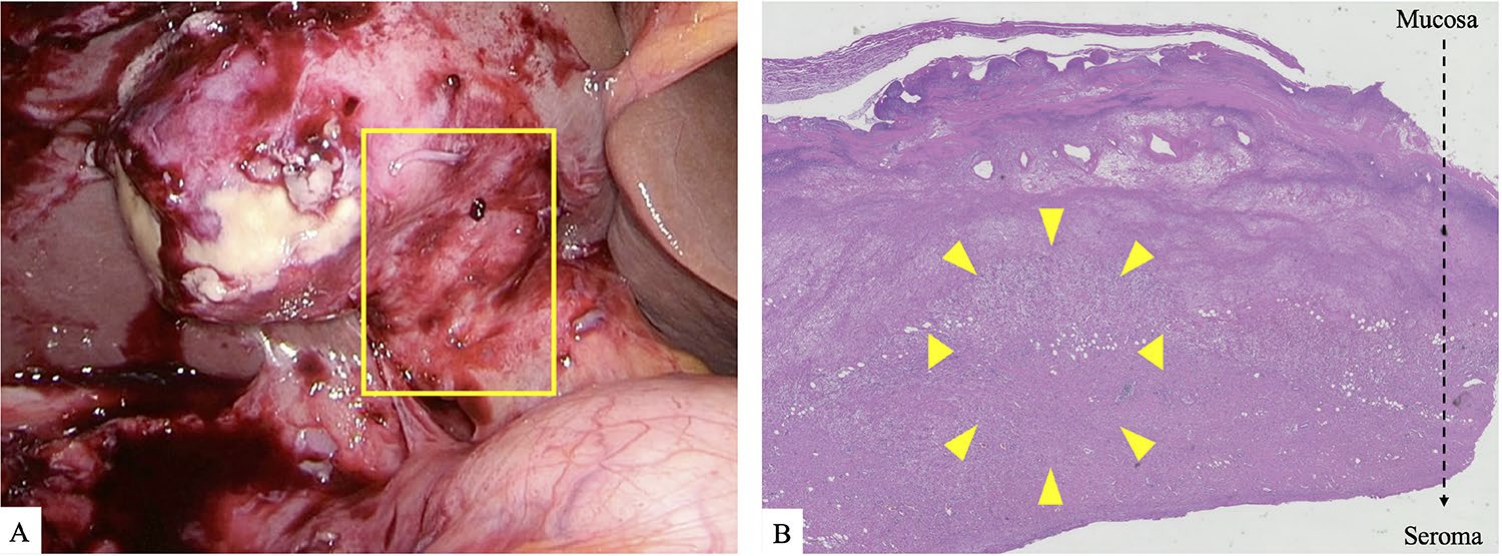
Example of dataset. **A** Laparoscopic images of intraoperative findings of scarring in gallbladder neck. **B** Scarring of gallbladder neck tissue (yellow arrow) in pathologic examination

**Fig. 2:**
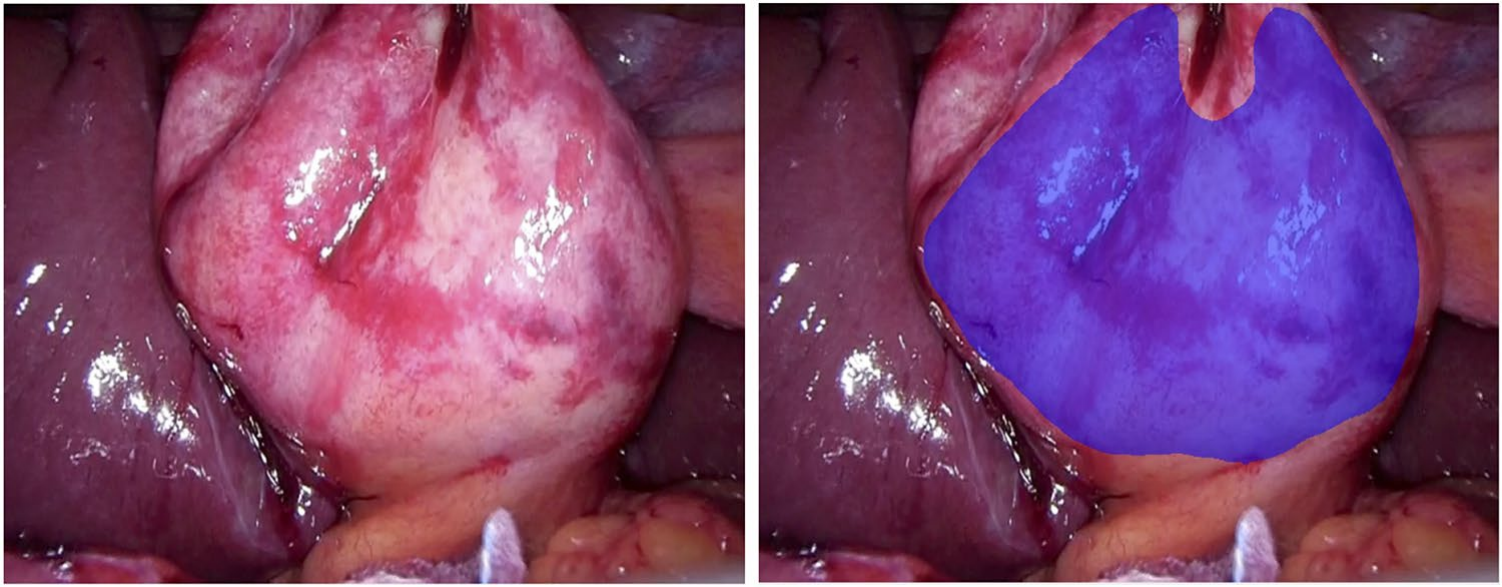
Example of annotation dataset for deep learning. Left: Laparoscopic image. Right: Annotation labels of the scarring area on laparoscopic images

Video data were collected from 158 LC cases of AC performed at Oita University between January 2010 and March 2024. Of these, 21 cases with intraoperative findings of scarring were used to develop the AI system. The characteristics of the cases used to develop the AI system are listed in Table 1. Anonymized video data of LC for AC performed at other centers were purchased from SurgStorage to evaluate the AI system. Of these, 20 cases with intraoperative scarring were included in this study to evaluate the AI system (Table 2). The data extracted from anonymized video data purchased from SurgStorage was only used to evaluate the Development AI system. Cases with partial and diffuse scarring in Calot’s triangle area were categorized as “grade B” and “grade C.” Of the 41 cases, 26 were grade B and 15 were grade C (Supplementary Fig. 1) [14]. Two surgeons, including at least one certified surgeon by the Endoscopic Surgical Skill Qualification System (ESSQS) developed by the Japan Society for Endoscopic Surgery (JSES), assessed the intraoperative findings after reviewing the surgical videos. Typical surgery video clips provided by the Japanese Society of Hepato-Biliary-Pancreatic Surgery, serving as diagnostic indicators of intraoperative findings, have been used to assess intraoperative scarring [15]. In the LC cases performed at our institution, histopathological examination confirmed the pathologic findings of scarring and ensured the diagnostic quality of the surgical findings (Fig. 1). We edited the videos into shorter segments showing scenes where the gallbladder and Calot’s triangle could be assessed. These short videos were used to create datasets for deep learning training and evaluation of the training model. Short videos were divided into still images. To create annotation datasets, surgeons annotated still images with scarred areas (Fig. 2). In this study, the scarring area was defined as the area that expert surgeons judged to be characteristic of the intraoperative findings of scarring on laparoscopic images. Annotation was performed by a board-certified surgeon from the Japan Surgical Society. Surgeons certified by the ESSQS from the JSES performed the final confirmation of annotation data.

**Table 1:** Characteristics of LC data for AI system development

Number	*n* = 21
Diagnosis	
Acute cholecystitis	21
Operation time (min)	214 ± 71
Blood loss (ml)	102 ± 108
Surgical difficulty grade	
Grade B	13
Grade C	8
Intraoperative findings	
Partial scarring of Calot’s triangle	13
Diffuse scarring of Calot’s triangle	8
Bailout surgery	8
Bile duct injury	0

*LC* Laparoscopic cholecystectomy, *AI* Artificial intelligence

**Table 2:** Characteristics of the data for evaluation

	Diagnosis	Operation time (min)	Blood loss (g)	Surgical difficulty grade	Intraoperative findings	Bailout Surgery
No.1	AC	120	50	Grade B	Partial scarring of Calot’s triangle	–
No.2	AC	120	350	Grade C	Diffuse scarring of Calot’s triangle	–
No.3	AC	210	50	Grade C	Diffuse scarring of Calot’s triangle	–
No.4	AC	150	100	Grade C	Diffuse scarring of Calot’s triangle	Fundus first
No.5	AC	120	50	Grade B	Partial scarring of Calot’s triangle	–
No.6	AC	120	150	Grade C	Diffuse scarring of Calot’s triangle	Fundus first
No.7	AC	180	200	Grade B	Partial scarring of Calot’s triangle	–
No.8	AC	120	0	Grade B	Partial scarring of Calot’s triangle	–
No.9	AC	120	100	Grade C	Diffuse scarring of Calot’s triangle	Fundus first
No.10	AC	150	100	Grade B	Partial scarring of Calot’s triangle	–
No.11	AC	90	100	Grade B	Partial scarring of Calot’s triangle	–
No.12	AC	180	350	Grade B	Partial scarring of Calot’s triangle	–
No.13	AC	180	400	Grade B	Partial scarring of Calot’s triangle	–
No.14	AC	90	50	Grade B	Partial scarring of Calot’s triangle	–
No.15	AC	180	0	Grade C	Diffuse scarring of Calot’s triangle	Fundus first, LSC
No.16	AC	240	0	Grade B	Partial scarring of Calot’s triangle	–
No.17	AC	210	0	Grade B	Partial scarring of Calot’s triangle	–
No.18	AC	240	0	Grade C	Diffuse scarring of Calot’s triangle	Fundus first
No.19	AC	210	0	Grade B	Partial scarring of Calot’s triangle	Fundus first, LSC
No.20	AC	240	0	Grade B	Partial scarring of Calot’s triangle	Fundus first

*AC* acute cholecystitis, *LSC* laparoscopic subtotal cholecystectomy

#### Deep learning model for scarring area detection

In this study, Hyperseg, an AI model of a semantic segmentation algorithm, was used to develop the AI [16]. The image resolution was resized to 416 × 416 pixels for image processing while building the training model. EfficientNet-B5, an image recognition algorithm, was used as the backbone [17]. The training class was set as a class of scarring areas. To improve the performance of the training model, data extensions such as rotation, resizing, and cropping were performed. A total of 2025 annotated data points from 21 cases at Oita University were prepared and used to train the AI.

### Evaluation of the AI system

Evaluating the developed AI system’s performance for real-time use during LC requires assessing both the accuracy and usefulness of the information provided and conducting a comprehensive clinical evaluation, including its effectiveness in preventing BDI. Therefore, the evaluation of the AI system developed in this study consisted of three evaluations: a quantitative evaluation of AI detection accuracy using still images, a qualitative evaluation of the AI system using surgical videos, and a questionnaire evaluation of the usefulness of AI systems. These evaluations were conducted by three certified surgeons from multiple institutions as an external evaluation committee (EEC) who did not belong to the research group to evaluate the AI system more objectively. The three surgeons in EEC were JSES certified by the ESSQS with 27, 21, and 17 years of clinical experience, respectively.

#### Quantitative evaluation of the AI detection accuracy using still images

Twenty surgical videos were used for the evaluation. Five still images of scenes in which intraoperative scarring findings could be identified from the surgical videos were extracted from each case, for a total of 100 still images used in the evaluation. The EECs annotated 100 still images using an annotation tool in scarred and compared the areas indicated by the EECs with those indicated by the AI to assess the accuracy of the AI system. The Dice coefficient was used to evaluate segmentation accuracy. These metrics can be described by Eqs. (1)–(3) below, where TP is a true positive, FP is a false positive, TN is a true negative, and FN is a false negative:

1 $${\text{Dice}}\;{\text{coefficient}} = {2} \cdot {\text{Precision}} \cdot {\text{Recall}}/\left( {{\text{Precision}} + {\text{Recall}}} \right),$$

2 $${\text{Precision}} = {\text{TP}}/\left( {{\text{TP}} + {\text{FN}}} \right),$$

3 $${\text{Recall}} = {\text{TP}}/\left( {{\text{TP}} + {\text{FN}}} \right).$$

#### Qualitative evaluation of an AI system using surgical videos

Although DICE coefficients are the standard method for evaluating domain extraction models, AI systems must be evaluated using videos because they are used for surgical videos in actual clinical practice. Therefore, we assessed the accuracy of the developed AI system using surgical videos. The EECs watched each video clip on two screens. The original video of the scene in which the gallbladder and gallbladder neck could be seen in LC was displayed on the left screen, and the video with the AI prediction superimposed was displayed on the right screen. The EECs watched video clips of 20 cases and answered questions on the AI detection accuracy in each case. The question, ‟How accurately does the AI-detected area of scarring correspond to your perception of the scarring area?” was posed on a five-point Likert scale (5 = Excellent, 4 = Good, 3 = Fair, 2 = Poor, and 1 = Fail) to evaluate the accuracy of AI detection. The intervals of the five-point Likert scale were defined in 20% increments (from a score of 5 for 80–100% success in detecting scarring areas to a score of 1 for 0–20% success in detecting scarring areas). Representative images of the original and AI predictions are shown in Supplementary Fig. 2.

#### Questionnaire evaluation of the utility of AI systems

After evaluating 20 cases, a questionnaire evaluating the utility of this AI system was administered. The questionnaire consisted of three clinical utility questions on AI’s usefulness in diagnosing surgical difficulty, making decisions regarding bailout surgery, and preventing BDI, and two educational utility questions on its usefulness in educating young surgeons and its usefulness in educating less experienced surgeons in medically undeveloped countries. The EECs responded on a five-point Likert scale (5 = Excellent, 4 = Good, 3 = Fair, 2 = Poor, and 1 = Fail) to assess the usefulness of the AI system. This single-center retrospective study was approved by the Ethics Committee of Oita University Hospital (#2663).

## Results

### Quantitative evaluation of the AI detection accuracy using still images

**Fig. 3:**
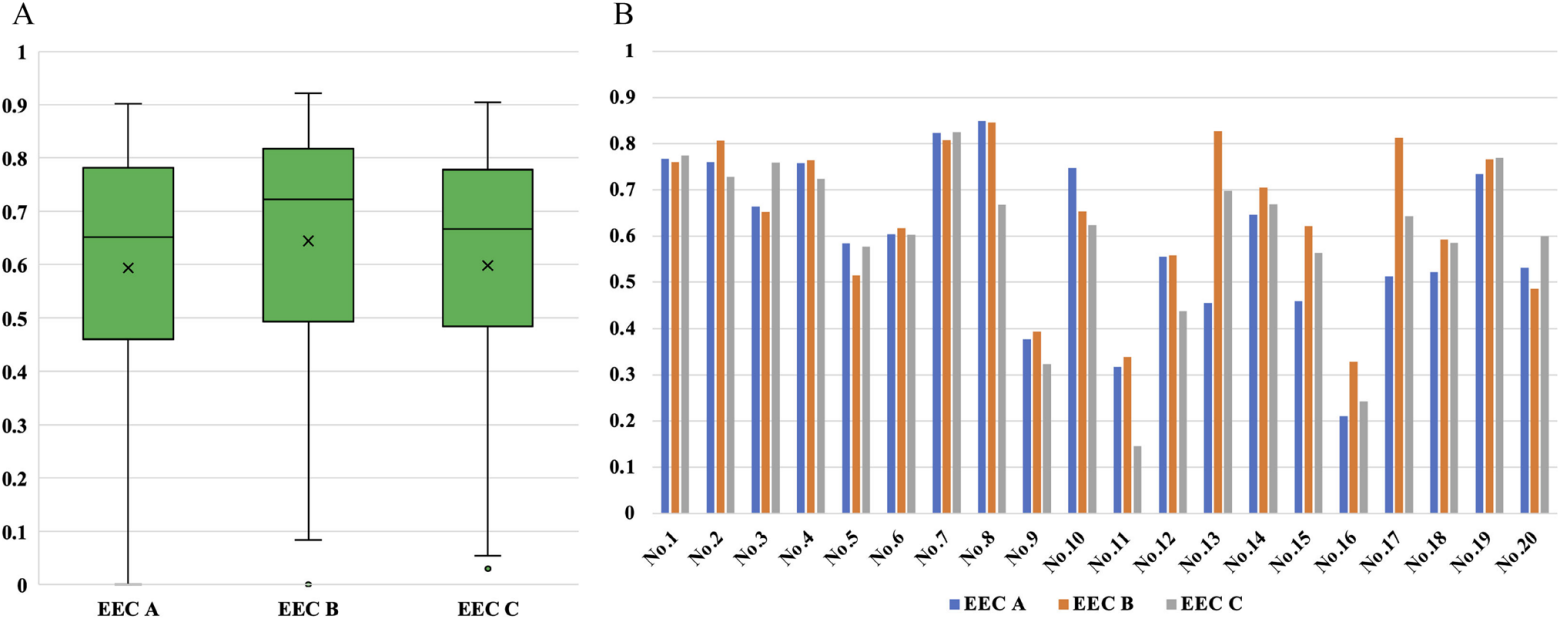
The results of quantitative evaluation. Results of the DICE coefficient between artificial intelligence detection and annotation by the external evaluation committee (EEC). Each EEC annotated the scarred area for five still images in each case. For 20 cases, 100 images were annotated using the EEC. **A** Overall DICE coefficient of the scarred area; **B** DICE coefficient for each case

**Fig. 4:**
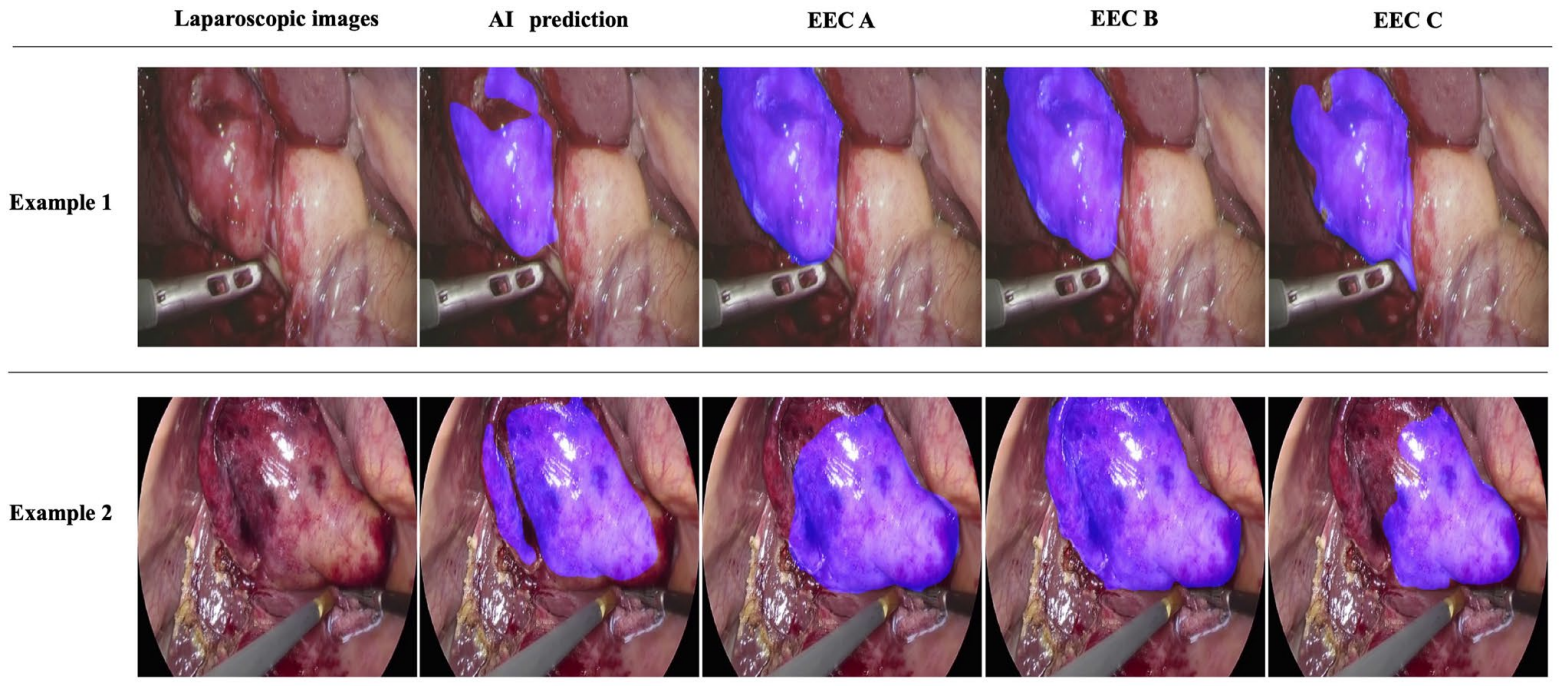
Representative examples of images showing good results in quantitative evaluation. The AI system was able to predict the area of scarring in each scene in the gallbladder region, including the Calot’s triangle; the AI system-predicted area and each annotated EEC were relatively consistent

**Fig. 5:**
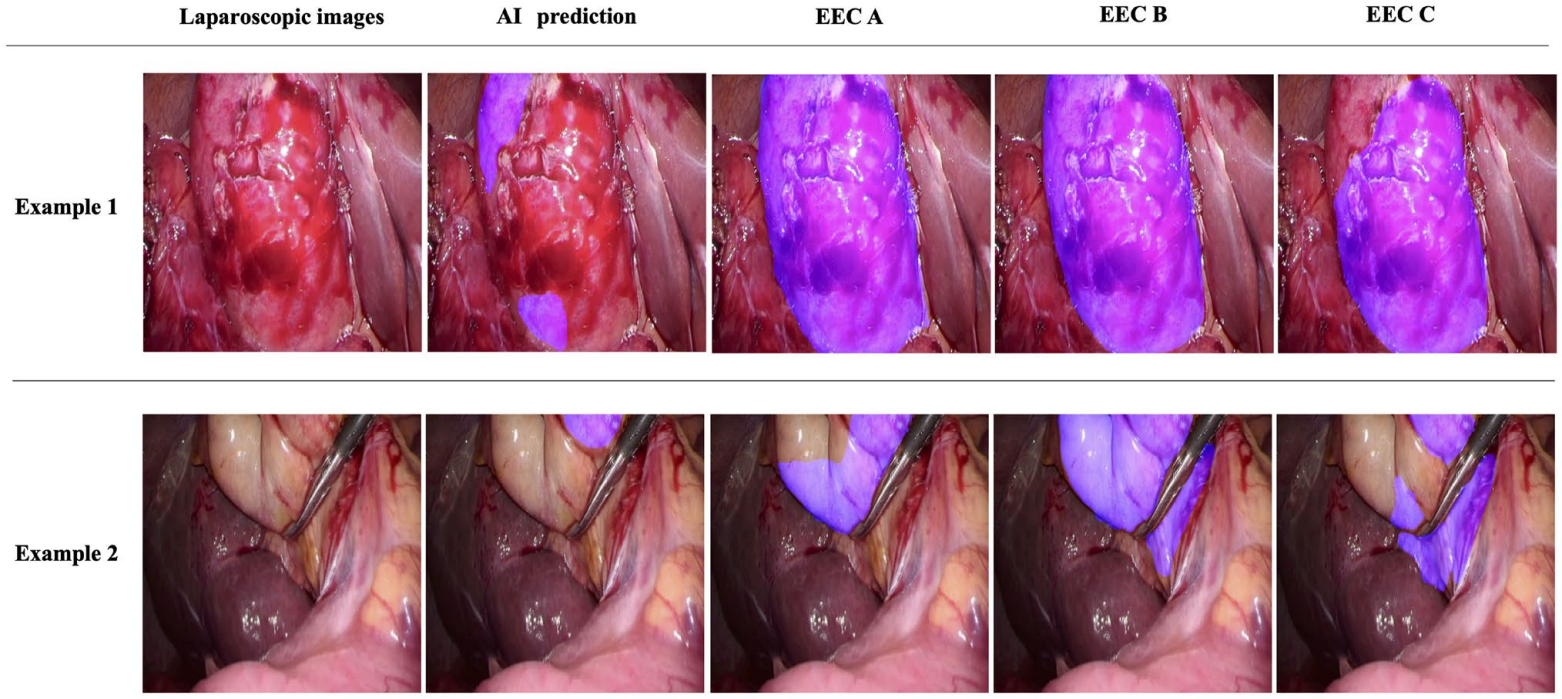
Representative examples of images showing poor results in quantitative evaluation. In scenes where blood contamination was present, such as in Example 1, the uncontaminated area could be predicted; however, the agreement with the area annotated by EEC was reduced. In cases with relatively mild scarring, such as in Example 2, the AI system did not adequately predict the scarred area, resulting in a low DICE coefficient

The Dice coefficients were calculated based on the annotation of the scarring area using EEC. Therefore, 100 still images from 20 cases were annotated by each EEC, and the DICE coefficient, precision, and recall for the scarred area were calculated. The average DICE coefficients for AI prediction of scarred areas and each EEC were 0.594 ± 0.245, 0.644 ± 0.235, and 0.598 ± 0.245 for EEC A, EEC B, and EEC C, respectively. The average overall DICE coefficient was 0.612 ± 0.241 (Fig. 3A). The average DICE coefficients for each evaluated case are shown in Fig. 3B. Case No. 7 resulted in the highest average DICE coefficient of 0.818 ± 0.076. Representative images of the AI detection results and EEC annotations are shown in Fig. 4. In these cases, the developed AI system could detect scarring areas in the gallbladder region, including the Calot’s triangle. This matched the high rate of scarred areas annotated by the EEC. By contrast, the average DICE coefficients were particularly low for cases 9, 11, and 16. Case 9 had Grade C operative difficulty with blood retention over the gallbladder, whereas Cases 11 and 16 had Grade B operative difficulty and a relatively mild degree of scarring. Representative images of scenes with low DICE coefficients are shown in Fig. 5.

### Qualitative evaluation of AI system using surgical videos

The results of the five-point evaluation of AI detection performance by EEC using surgical videos are presented in Table 3. The overall mean of the evaluation was 3.98 ± 0.76. In the evaluation of each case, the AI detection performance in case No. 12 had the highest evaluation (4.67 ± 0.47), with an average evaluation of 4 or more points in 15 of the 20 cases.

**Table 3:** Qualitative evaluation scores using surgical videos for each case

	EEC A	EEC B	EEC C	Average
No.1	4	4	4	4.00 ± 0.00
No.2	4	4	5	4.33 ± 0.47
No.3	4	4	4	4.00 ± 0.00
No.4	4	5	4	4.33 ± 0.47
No.5	4	4	5	4.33 ± 0.47
No.6	4	3	5	4.00 ± 0.82
No.7	4	4	4	4.00 ± 0.00
No.8	3	4	5	4.00 ± 0.82
No.9	3	4	5	4.00 ± 0.82
No.10	3	3	2	2.67 ± 0.47
No.11	1	4	5	3.33 ± 1.70
No.12	4	5	5	4.67 ± 0.47
No.13	3	4	4	3.67 ± 0.47
No.14	3	4	5	4.00 ± 0.82
No.15	4	4	5	4.33 ± 0.47
No.16	4	3	4	3.67 ± 0.47
No.17	4	4	4	4.00 ± 0.00
No.18	4	4	5	4.33 ± 0.47
No.19	3	4	4	3.67 ± 0.47
No.20	4	4	5	4.33 ± 0.47
				3.98 ± 0.76

*EEC* external evaluation committee

### Questionnaire evaluation of the utility of AI systems

The questionnaire results are shown in Table 4. The ratings of EEC in the clinical usefulness of the developed AI system were 4.33, 4.67, and 4.33 for assistance in the diagnosis of surgical difficulty, assistance in decision-making for avoidable surgery, and effectiveness in the prevention of BDI, respectively. The rating of EEC in terms of usefulness in education was 4.67 for both young surgeons and surgeons from medically underdeveloped countries. These results demonstrate the potential clinical and educational utility of AI systems.

**Table 4:** Results from the AI system's questionnaire evaluation

	EEC A	EEC B	EEC C	Average
Surgical difficulty	4	4	5	4.33 ± 0.47
Bailout surgery	4	5	5	4.67 ± 0.47
To prevent BDI	4	5	4	4.33 ± 0.47
Young surgeon	4	5	5	4.67 ± 0.47
Surgeon in medically undeveloped countries	4	5	5	4.67 ± 0.47

*AI* artificial intelligence, *EEC* external evaluation committee, *BDI* bile duct injury

## Discussion

We developed an AI-based real-time detection system for intraoperative scarring and evaluated it using EEC cases from other centers. In a quantitative evaluation of the AI detection performance using still images, the average DICE coefficient for scarred areas annotated by the EEC members and detected by the AI was 0.612. In a qualitative evaluation of the AI detection performance using surgical videos, EEC members generally gave high scores on a five-point scale. Questionnaire evaluation also showed that certified surgeons who were not involved in its development recognized the usefulness of the AI system in clinical practice and education. AI technology is rapidly spreading to various surgical fields worldwide and is expected to contribute to improving surgical outcomes and the quality of education, and equalizing healthcare disparities [18]. The development of surgical support systems for LCs using AI has also been reported [19, 20]. However, few reports exist on AI systems assisting LC for AC, which present a variety of findings depending on the degree of inflammation and varying degrees of surgical difficulty. Ward et al. developed and reported an AI system to identify the degree of inflammation in the gallbladder [21]. This system enables the AI to identify the degree of inflammation in AC based on the Parkland grading system (PGS) [22] and predict the operative course of LC for AC, thus enabling the AI to diagnose the surgical difficulty of AC. However, PGS lacks information on Calot’s triangle, which is associated with the risk of BDI in more severe AC. The Tokyo guidelines 18 included 25 intraoperative findings as indicators for real-time, objective intraoperative assessment of surgical difficulty [13]. Asai et al. developed a surgical difficulty assessment system based on these intraoperative findings [14]. This surgical difficulty grading system examined the effects of inflammation in AC in more detail, including Calot’s triangle. Of these, scarring is a relatively common and critical finding for the risk of BDI and is most likely to be encountered in practice. Furthermore, diagnosis of scarring is crucial information to determine the need for bailout surgery to prevent BDI, as well as recognition of the anatomic structure of bile duct. In this study, we developed an AI system to intraoperatively indicate the same scarred areas as those determined by expert surgeons in LC for AC. The findings from the development of our AI system and the evaluation by EEC showed the potential for new surgical AI systems in LC for AC. A quantitative evaluation of the AI detection accuracy using still images resulted in an overall average DICE coefficient of 0.612. This value was comparable to previous reports identifying anatomic structures in intraoperative videos, such as the identification of a safe zone of dissection in laparoscopic cholecystectomy (0.70) and nerve detection in laparoscopic colon resection (0.56) [19, 23]. In the quantitative evaluation of each case, the average DICE coefficient was around 0.7 in many cases with a maximum average DICE coefficient of 0.818. Furthermore, in the qualitative evaluation using videos, the developed AI was able to sufficiently detect scarring areas in the gallbladder region, including the Calot’s triangle, which was the initial objective in the surgical videos, and showed relatively comparable teaching performance to EEC recognition in many cases of surgical videos (Supplementary Video). This AI system indicated high accuracy that did not deviate from the EEC’s recognition; furthermore, it would be a general system that could be applied to LC in other institutions because the data for evaluation were collected from other centers and evaluated by the EECs. However, bleeding resulted in a low DICE coefficient in the quantitative evaluation using still images. Quantitative evaluation using still images showed that the DICE coefficient was low, as expected, because the area where the scarring area could be recognized was smaller in scenes where contamination of the surgical field by blood was noticeable, whereas quantitative evaluation using videos showed good indication performance in the same scenes where the scarring area was detected by avoiding the blood-contaminated area. In some cases of partial scarring of Calot’s triangle, the quantitative evaluation showed a low DICE coefficient, and the qualitative evaluation tended to be inaccurate. These patients exhibited relatively small scarring areas. Insufficient study data for cases with a small area of scarring was suggested as the reason for the lower accuracy. To ensure high accuracy in these cases, a larger training dataset including milder scars is required. Based on these AI performance evaluations, the questionnaire also showed that EEC rated the AI system higher for clinical usefulness, including diagnosing surgical difficulty, deciding to perform bailout surgery, and preventing BDI. The results showed that EEC found this information useful for the prevention of BDI during LC in AC. The usefulness of the system for less-experienced surgeons was also evaluated. In our previous study, we developed an AI system to indicate important anatomical landmarks on another screen during LC to prevent BDI and reported that the AI system indication affected the surgeon’s recognition of landmarks [24]. The AI system developed in this study could also be desirable for assisting in the diagnosis of intraoperative scarring by influencing the perception of less experienced novice surgeons. In addition, the AI system developed in this study could be combined with our AI system indicating anatomical landmarks and ICG cholangiography showing of the common bile duct anatomy, potentially improving its effectiveness in preventing BDI.

This study attempted to detect detailed intraoperative findings using AI by visualizing expert recognition of intraoperative findings in acute cholecystitis and found it to be technically feasible. Recognition of intraoperative findings is important not only in acute cholecystitis but also in other areas of surgery where tacit knowledge is required to recognize appropriate intraoperative findings. We demonstrated that the possibility of developing an AI system to detect intraoperative findings that have already been established as important in AC and demonstrated the clinical utility of this system. In other surgical procedures, the detection of intraoperative findings is important for safe surgery and requires expert surgeons to acquire tacit knowledge. We hope that the AI system for detecting intraoperative findings proposed in this study can be applied to other surgical procedures to prevent complications and improve surgical outcomes. In future, the AI system technology we are developing is also expected to deliver results in robotic surgery. Robotic surgery, which demands enhanced visual information compared to laparoscopic surgery, could greatly benefit from the AI systems we are developing. This study has several limitations. The data used for deep learning were small and from a single center, and the annotation of scarring areas relied on a limited number of annotators. Since cases were carefully selected to improve the quality of the training data, the number of cases used for training was relatively small. Additionally, only 20 cases of data were available for evaluation, and the number of assessors was small. Further studies with larger-scale datasets to improve performance, as well as evaluation and comparisons of the diagnostic accuracy between the AI system and surgeons, are needed to develop a system that can be used clinically in future. However, this study demonstrates the feasibility of an AI system for detecting intraoperative findings of AC and highlights its clinical value. The AI system developed in this study is expected to further prevent BDI in LC for AC with improved performance and in combination with other systems showing anatomic structures. In conclusion, we successfully developed an AI system for real-time detection of intraoperative scarring at LC in AC. By assisting in the recognition of intraoperative findings related to surgical difficulty, this system is expected to improve surgical safety and prevent BDI. Further performance improvements and validation are needed for clinical use in future.

## Supplementary Information

Below is the link to the electronic supplementary material.

Supplementary file1 (DOCX 1235 kb)

Supplementary file2 (MP4 87601 kb)
