# Supplementary material for: Development of an artificial intelligence system to indicate intraoperative findings of scarring in laparoscopic cholecystectomy for cholecystitis
Source: Surg Endosc. 2025 Jan 21;39(2):1379–87. doi: 10.1007/s00464-024-11514-2 (PMC11794413; doi:10.1007/s00464-024-11514-2)
Supplement: Supplementary file 1 — Supplementary file1 (DOCX 1235 kb) [file 464_2024_11514_MOESM1_ESM.docx]

**Supplementary figure 1 Surgical difficulty grading system based on 25 intraoperative findings.**

The higher the score, the more difficult the surgery, and the higher the risk of BDI.

Consideration of bailout surgery is recommended in cases classified as Grade C.


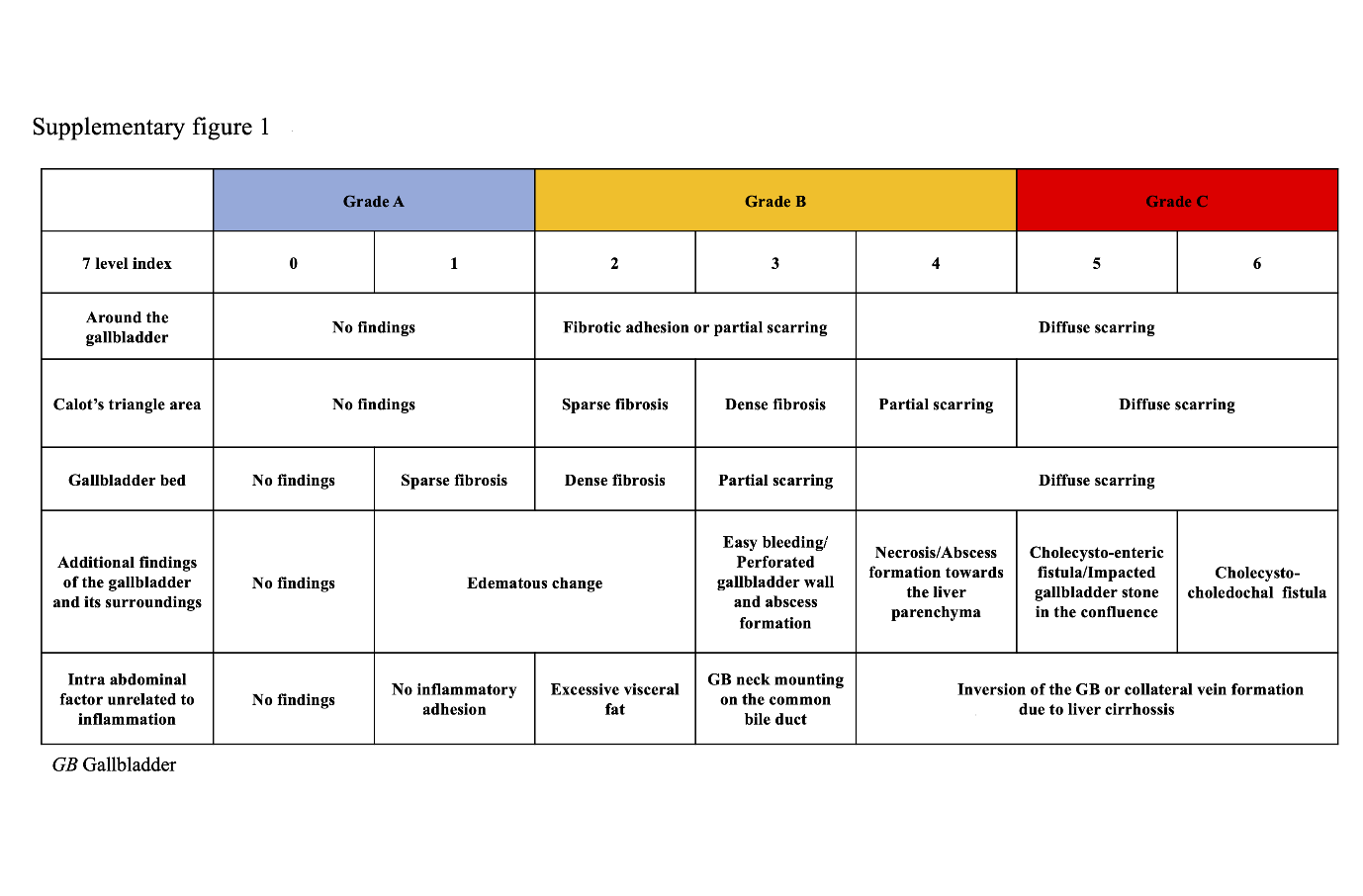


**Supplementary figure 2 Representative captured images of original and prediction clips in qualitative evaluation using surgical videos**
